# Complete chloroplast genome sequence and phylogenetic analysis of *Annona reticulata*

**DOI:** 10.1080/23802359.2020.1829131

**Published:** 2020-10-09

**Authors:** Ying-feng Niu, Kai-Xiong Li, Jin Liu

**Affiliations:** Yunnan Institute of Tropical Crops, Jinghong, China

**Keywords:** *Annona reticulata*, chloroplast genome, phylogenetic relationship

## Abstract

*Annona reticulata* is native to South and Central America which has many phytochemical and pharmacological activities suggesting a wide range of clinical application in lieu of cancer chemotherapy. This study provides abundant genomic data for the genetic relationship study, germplasm resources evaluation and varieties selection of *A. reticulata*. The complete chloroplast genome of *A. reticulata* was sequenced, assembled, and annotated in this study. The genome size was 201,906 bp and was divided into four regions: a large single-copy region of 69,650 bp, a small single-copy region of 3,014 bp, and two inverted repeat regions of 64,621 bp. A total number of 164 genes were annotated, including 115 protein-coding genes, one pseudogene, 40 tRNA genes, and eight rRNA genes. In terms of gene function, the 164 genes were divided into four major groups: genes for self-replication, photosynthesis, unknown function, and other genes. A maximum likelihood tree based on the chloroplast genome sequences of 24 plant species was constructed. The result of phylogenetic analysis showed that *A. cherimola* had the closest relationship with *A. reticulate*.

*Annona reticulata* is a medium sized plant, native to South and Central America, belonging to the Annonaceae family (Mondal et al. 2008). The *Annona* genus consists of about 119 species, most of which are shrubs and trees, and are widely distributed in the tropical and subtropical regions (Thang et al. 2013). The fruit of *A. reticulata* has a smooth skin, which becomes dull red when ripe. It is commonly known as bullock’s heart or custard apple. Its custard like pulp is rather sugary and less flavored as compared to other familiar species, so it’s usually eaten as a dessert fruit and made into drinks and ice creams (Jorge et al. 2003; Ogunwande et al. 2006). Besides, *A. reticulate* has been used as a traditional medicine in some of the southeast Asian countries (Mondal et al. 2007), such as India, Malaysia, Indonesia, Thailand, Vietnam and some other countries. The phytochemical and pharmacological activities of *A. reticulata* components suggest a wide range of clinical applications in lieu of cancer chemotherapy (Sureshet al. 2011), and it is having various other pharmacological activities, such as antioxidant, analgesic and CNS depressant, antimalarial, anthelmintic, syphilis, and few more (Chavan 2014).

In the recent times, many studies have focused on the isolation, characterization and utilization of natural antioxidants of *A. reticulata* (Suneelkumar et al. 2011). However, there has been little research on its genome. The chloroplast genome is a relatively independent genetic system in the plant cells that contains abundant genetic information, which encodes many genes relating to photosynthesis and other important biological processes. Research on chloroplast genome is very important for analysis of plant evolution, genetic relationship identification and germplasm resource evaluation. In this study, the complete chloroplast genome of *A. reticulata* was sequenced, assembled, and annotated.

The specimen of *A. reticulata* was collected from from the Xishuangbanna Tropical Flowers and Plants Garden (100.70422 E, 22.015885 N) and deposited in the herbarium of Yunnan Institute of Tropical Crops (Xishuangbanna, China) with the specimen voucher number of YITC-2020-FZ-A-004. The genomic DNA extraction was done by using the Dneasy Plant Mini Kit (Qiagen). DNA sample quality and quantity were characterized by gel electrophoresis and Nano-Drop 2000 spectrometer (Thermo Fisher Scientific, USA). The high-quality genomic DNA were used to prepare DNA library following the manufacturer's instructions (Illumina, San Diego, CA) with insert sizes of 350 bp for paired-end sequencing, paired-end (PE) sequencing was conducted on the Illumina Hiseq 2500 Platform (Illumina, San Diego, CA). The chloroplast genome of *A. reticulata* was assembled by CLC Genomics Workbench v3.6 (http://www.clcbio.com) and annotated by DOGMA (Wyman et al. 2004), using the cp annotation of *Annona cherimola* (NC_030166) as a reference, and then uploaded to the GenBank (http://www.ncbi.nlm.nih.gov/) with the accession number MT742547.

The complete chloroplast genome of *A. reticulate* is double stranded, circular structure with the size of 201,906 bp, and is composed of 61,242 A bases (30.33%), 60,797 T bases (30.11%), 40,426 G bases (20.02%), and 39,441 C bases (19.53%). The GC content of the chloroplast genome is 39.55%. Like other plants, the chloroplast genome of *A. reticulate* consists of four regions: a large single-copy region (LSC, 69,650 bp) and a small single-copy region (SSC, 3,014 bp), separated by two inverted repeat regions (IRa and IRb, 64,621 bp). A total number of 164 genes were annotated, include 115 protein-coding genes (PCGs), one pseudogene, 40 tRNA genes, and eight rRNA genes. In terms of gene function, 164 genes are divided into four major groups: genes for self-replication, photosynthesis, unknown function and other genes. There are 46 genes involved in photosynthesis that are divided into six groups: Subunits of NADH dehydrogenase (*ndhA*, *ndhB*, *ndhC*, *ndhD*, *ndhE*, *ndhF*, *ndhG*, *ndhH*, *ndhI*, *ndhJ*, *ndhK*), Large subunit of Rubisco (*rbcL*), Subunits of photosystem II (*psbA*, *psbB*, *psbC*, *psbD*, *psbE*, *psbF*, *psbH*, *psbI*, *psbJ*, *psbK*, *psbL*, *psbM*, *psbN*, *psbT*, *psbZ*), Subunits of photosystem I (*psaA*, *psaB*, *psaC*, *psaI*, *pasJ*, *ycf3*, *ycf4*), Subunits of ATP synthase (*atpA*, *atpB*, *atpE*, *atpF*, *atpH*, *atpI*), and Subunits of cytochrome (*petA*, *petB*, *petD*, *petG*, *petL*, *petN*).

A maximum likelihood tree based on the chloroplast genome sequences of 24 plant species was constructed to study the phylogenetic relationship between *A. reticulata* and other plant species (Figure 1). The jModelTest 2.1.7 (David 2008) software was employed to analyze nucleotide substitutions model under the Akaike Information Criterion (AIC), the GTR + G + I model was selected for nucleotide and the phylogenetic analysis was carried out using the maximum likelihood method with the RAxML8.1 (Alexandros 2006). Statistical supports were assessed with 1000 bootstrap replicates. Out of 24 plant species, 17 species belonged to Magnoliaceae family, five to Annonaceae family, one to Myristicaceae family and *Trochodendron aralioides* belonged to Trochodendraceae family, which was used as the out group. Multiple sequence alignment was carried out by MAFFT (Katoh and Standley 2013) and maximum-likelihood (ML) analysis was carried out by MEGA7.0 (Kumar et al. 2016). Phylogenetic analysis indicated that *Annona cherimola* had the closest relationship with *A. reticulata.* This study provides abundant genomic data for the genetic relationship study, germplasm resources evaluation and varieties selection of *A. reticulata*.

**Figure 1.**
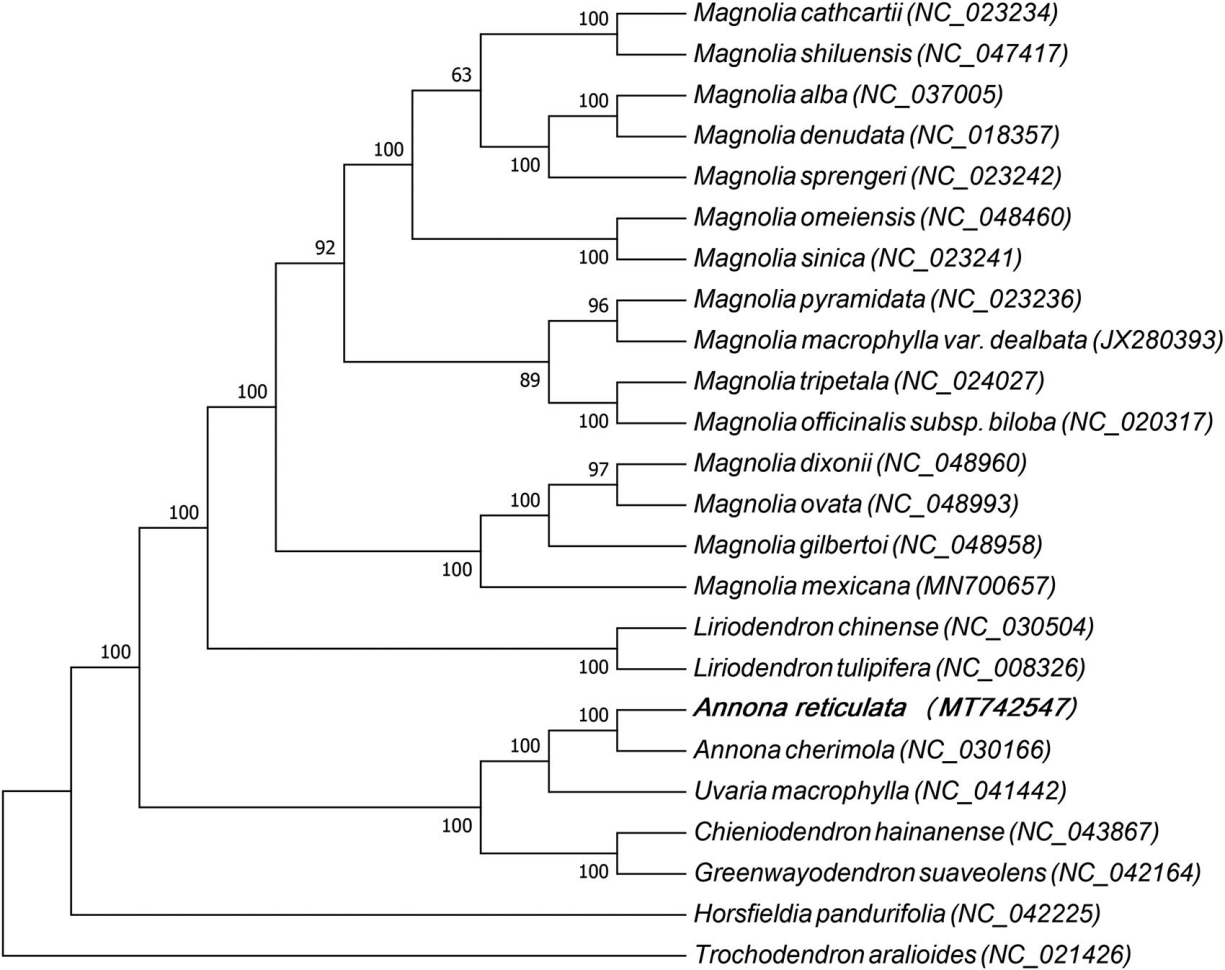
Phylogenetic tree based on the chloroplast genome sequences of 24 plant species, of which 17 species belong to Magnoliaceae family, five to Annonaceae family, one to Myristicaceae family and *Trochodendron aralioides* belongs to Trochodendraceae family which isused as the out group. The species and GenBank accession numbers of the 24 chloroplast genomes for the ML tree construction has also been mentioned in Figure 1. *Magnolia cathcartii* (NC_023234), *Magnolia shiluensis* (NC_047417), *Magnolia alba* (NC_037005), *Magnolia denudata* (NC_018357), *Magnolia sprengeri* (NC_023242), *Magnolia omeiensis* (NC_048460), *Magnolia sinica* (NC_023241), *Magnolia pyramidata* (NC_023236), *Magnolia macrophylla var. dealbata* (JX280393), *Magnolia tripetala* (NC_024027), *Magnolia officinalis subsp. biloba* (NC_020317), *Magnolia dixonii* (NC_048960), *Magnolia ovata* (NC_048993), *Magnolia gilbertoi* (NC_048958), *Magnolia mexicana* (MN700657), *Liriodendron chinense* (NC_030504), *Liriodendron tulipifera* (NC_008326), *Annona reticulata* (MT742547), *Annona cherimola* (NC_030166), *Uvaria macrophylla* (NC_041442), *Chieniodendron hainanense* (NC_043867), *Greenwayodendron suaveolens* (NC_042164), *Horsfieldia pandurifolia* (NC_042225), *Trochodendron aralioides* (NC_021426).

## Data Availability

The chloroplast genome sequence data that support the findings of this study are openly available in GenBank at https://www.ncbi.nlm.nih.gov/, reference number MT742547. The raw sequencing data are openly available in SRA database with the accession number PRJNA658442 and SRR12506405.

## References

[CIT0001] Alexandros S. 2006. RAxML-VI-HPC: maximum likelihood-based phylogenetic analyses with thousands of taxa and mixed models. Bioinformatics. 22(21):2688–2690. 16928733 10.1093/bioinformatics/btl446

[CIT0002] Chavan SS. 2014. A comprehensive review on *Annona reticulata*. J Pharm Sci Res. 5(1):45–50.

[CIT0003] David P. 2008. jModelTest: phylogenetic model averaging. Mol Biol Evol. 25(7):1253–1256. 18397919 10.1093/molbev/msn083

[CIT0004] Jorge AP, Rolando M, Victor F. 2003. Characterization of volatiles in bullock's heart (*Annona reticulata* L.) fruit cultivars from Cuba. J Agric Food Chem. 51(13):3836–3839. 12797752 10.1021/jf020733y

[CIT0005] Katoh K, Standley DM. 2013. MAFFT multiple sequence alignment software version 7: improvements in performance and usability. Mol Biol Evol. 30(4):772–780. 23329690 10.1093/molbev/mst010 PMC3603318

[CIT0006] Kumar S, Stecher G, Tamura K. 2016. MEGA7: molecular evolutionary genetics analysis version 7.0 for bigger datasets. Mol Biol Evol. 33(7):1870–1874. 27004904 10.1093/molbev/msw054 PMC8210823

[CIT0007] Mondal S, Mondal N, Mazumder U. 2007. In vitro cytotoxic and human recombinant caspase inhibitory effect of *Annona reticulata* leaves. Indian J Pharmacol. 39(5):253–254.

[CIT0008] Mondal SK, Saha P, Mondal N, Mazumder UK. 2008. Free radical scavenging property of *Annona reticulata* leaves. Orient Pharm Exp Med. 8(3):260–265.

[CIT0009] Ogunwande IA, Ekundayo O, Olawore NO, Kasali AA. 2006. Essential oil of *Annona reticulata* L. leaves from Nigeria. J Essent Oil Res. 18(4):374–376.

[CIT0010] Suneelkumar A, Venkatarathanamma V, Suneeta K, Kumari B. 2011. Comparative in vitro screening of α-Amylase and α-Glucosidase enzyme inhibitory studies in leaves of *Annona* species. J Pharm Res. 4(12):4431–4434.

[CIT0011] Suresh H, Shivakumar B, Hemalatha K, Heroor S, Hugar D, Sambasiva Rao K. 2011. In vitro antiproliferativeactivity of *Annona reticulata* roots on human cancer cell lines. Pharmacognosy Res. 3(1):9–12. 21731389 10.4103/0974-8490.79109 PMC3119276

[CIT0012] Thang TD, Kuo PC, Huang GJ, Hung NH, Huang BS, Yang ML, Luong NX, Wu TS. 2013. Chemical constituents from the leaves of *Annona reticulata* and their inhibitory effects on NO production. Molecules. 18(4):4477–4486. 23591927 10.3390/molecules18044477 PMC6270106

[CIT0013] Wyman SK, Jansen RK, Boore JL. 2004. Automatic annotation of organellar genomes with DOGMA. Bioinformatics. 20(17):3252–3255. 15180927 10.1093/bioinformatics/bth352

